# Evidence for the benefit of prenatal pelvimetry in breech presentation at term: A low intertuberous distance is associated with assisted head delivery and short‐term neonatal morbidity

**DOI:** 10.1002/ijgo.70780

**Published:** 2026-01-01

**Authors:** Julia Schmidt, Anna Elisabeth Hentrich, Anne Kristina Kämpf, Eileen Deuster, Samira Catharina Hoock, Frank Louwen, Lukas Jennewein

**Affiliations:** ^1^ Department of Obstetrics and Perinatal Medicine, Goethe‐University Hospital Goethe University Frankfurt Frankfurt am Main Germany

**Keywords:** breech delivery, intertuberous distance, prenatal pelvimetry

## Abstract

**Objective:**

Guidelines on vaginal breech delivery have several restrictions concerning feto‐maternal parameters. To date, they neglect the impact of prenatal pelvimetry, especially of the intertuberous distance (ITD), in nulliparous women attempting vaginal breech birth.

**Methods:**

We performed a prospective cohort study involving 876 nulliparous women with a breech presentation at term, analyzing the impact of the ITD measured with magnetic resonance imaging (MRI) imaging prior to delivery on maternal and fetal short‐term outcome parameters, as well as the necessity for manual assistance during vaginal breech delivery. Bivariate linear and nominal regression as well as multivariate adjusted nominal‐logistic regression with Walds‐testing was performed.

**Results:**

We found a significant negative correlation between the ITD and fetal short‐term morbidity (odds ratio [OR] per cm 0.56, 95% confidence interval [CI]: 0.31–0.98, *P* = 0.048). Significance was not reached when excluding deliveries with an ITD of below 11 cm. Multivariate adjusted regression showed a significant correlation of cesarean section and ITD (OR 0.72, 95% CI: 0.62–0.85, *P* < 0.001). In a multivariate subcohort analysis of all vaginal deliveries, the ITD was significantly associated with assisted head delivery (OR 0.67, 95% CI: 0.54–0.83, *P* < 0.001) and fetal short‐term morbidity (OR 0.44, 95% CI: 0.20–0.90, *P* = 0.031) when adjusted to fetal birth weight, head circumference and the obstetrical conjugate (OC). When cases with an ITD of below 11.5 cm were excluded, no significant association of the ITD and fetal short‐term morbidity was detected.

**Conclusion:**

Prenatal pelvimetry in breech presentation at term can be used to select patients for vaginal birth attempts. Women with an intertuberous distance (ITD) of below 11 cm might benefit from a cesarean section recommendation.

## 1 INTRODUCTION

At the end of pregnancy, 3%–4% of mothers expect infants in breech presentation and seek counseling regarding the safest delivery mode.^1^ Most obstetricians would recommend elective cesarean section based on the lack of clinical knowledge. According to current guidelines, the vaginal delivery out of breech presentation is considered a safe option and should be offered to selected women.^2^, ^3^ Nevertheless, breech presentation at term is among the leading causes for elective cesarean section, which leads to increased maternal and fetal morbidity worldwide.^4^ However, several studies have demonstrated that with a cautious selection of patients and a skilled obstetric team, long‐term maternal and fetal outcomes are not worse than those of a planned cesarean section, mainly when women deliver in an upright position.^5^, ^6^, ^7^, ^8^, ^9^, ^10^, ^11^, ^12^, ^13^, ^14^, ^15^, ^16^, ^17^, ^18^, ^19^ Therefore, more refined selection criteria are necessary to determine the eligibility of women for a trial of labor in cases of breech presentation. Shared decision making based on the current evidence should be the ambition in counseling women when selecting the women's preferred delivery mode. Currently, there is insufficient data on pelvic measurements and their predictive value for birth outcomes to be incorporated into guideline recommendations. Previous studies have examined the obstetrical conjugate (OC) and found it to be associated with cesarean section when measured low, for example, less than 12 cm.^12^, ^14^, ^20^, ^21^ Further, the interspinous distance (ISD) was examined to play a role in birth mechanics in breech deliveries.^22^ A study by Lia et al. on 268 deliveries showed a correlation of a low intertuberous distance (ITD) with obstetrical maneuvers, documenting an essential role of the ITD on breech birth mechanics.^23^ Due to the limited evidence on the ITD and its impact on the outcome of vaginal breech deliveries, we performed a prospective cohort study involving 876 nulliparous women to assess the impact of the ITD on delivery outcome, maternal and fetal short‐term outcome parameters, as well as the necessity for manual assistance during vaginal breech birth.

## 2 MATERIALS AND METHODS

### 2.1 Patient selection and ethics committee approval

Between January 2007 and December 2022, we conducted a prospective monocentric cohort study on vaginally intended deliveries of term singleton breech pregnancies (>37 weeks of pregnancy) in nulliparous women at the Goethe University Hospital in Frankfurt. This study was performed in accordance with the current version of the Helsinki Declaration.

The exclusion criteria for this study included an estimated fetal birth weight below 2500 g, multiple pregnancies, patients with inadequately managed diabetes or complications affecting the mode of delivery, as well as the mother's wish for a planned cesarean section. All women with breech presentations come to our center for counseling before birth to discuss the advantages and risks of planned vaginal delivery and cesarean section. They are offered an external version as a routine procedure. Standard clinical care is applied to all study patients. Vaginal breech delivery is mainly carried out in an upright birth position, for example, on hands and knees, squatting, kneeling, or standing, as in these positions the ITD enlarges in contrast to the supine position.^24^, ^25^ During delivery in an upright position, manual assistance is not mandatory and is only performed if necessary: For impacted arms the Louwen maneuver in an upright position and the Frank Nudge for delayed head delivery.^5^, ^13^ From the publication date of the study by Klemt et al., patients with an ITD of below 11 cm were recommended a cesarean section because of the risk for delayed fetal head delivery and the high risk for cesarean section as an individual clinical guideline.^12^ The university clinic's ethics committee authorized this study as a prospective registry study (2021‐126) with retrograde data analysis of all cases prior to the committee's vote. Subsequently, all patients provided written consent before birth for the scientific use of their anonymized data. The study is registered in the German clinical study registry (DRKS00025030).

### 2.2 Magnetic resonance imaging (MR) pelvimetry

**FIGURE 1:**
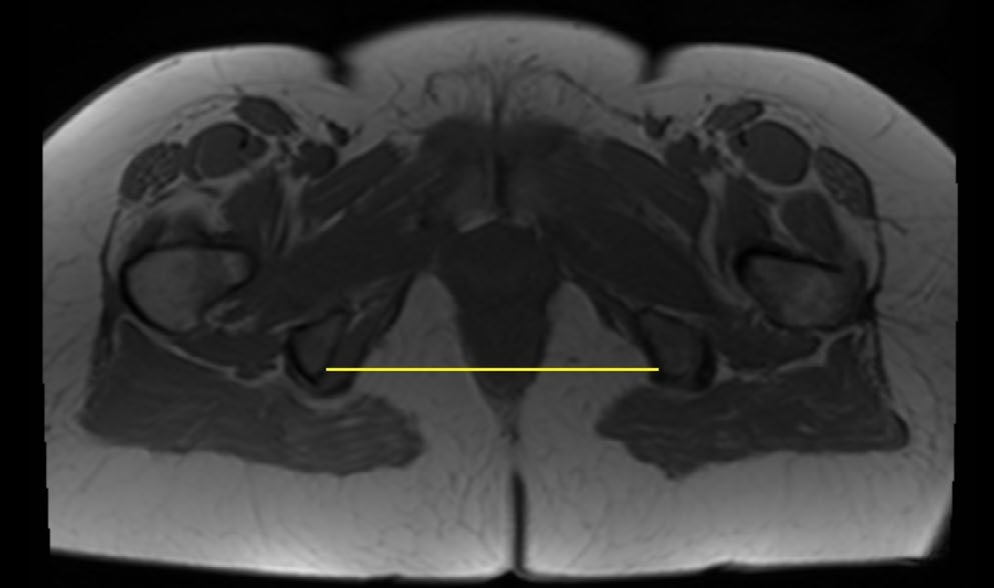
Magnetic resonance imaging (MRI) image with marked intertuberous distance (yellow line).

Data were collected using the hospital's patient management system. The ITD is defined as the transversal distance between the inner edge of both ischial tuberosities and was measured in a transversal image of the pelvic MRI. A measurement example is shown in Figure 1. Imaging occurred between 35 and 38 weeks of gestation in the supine position. All nulliparous women received an MRI of the pelvis during counseling in our center. A specialized radiologist evaluated the imaging. Measurements of pelvic distances, such as the ITD, were performed repeatedly by the study supervisors for quality assurance purposes and corrected by the supervisors in case of discrepancy.

### 2.3 Outcome parameters

Primary outcome parameters were fetal short‐term morbidity (PREMODA score) and cesarean section. In the subgroup analysis of vaginal deliveries, primary outcome parameters were fetal short‐term morbidity and assisted arm and head delivery.

The adapted Presentation et Mode d'Accouchement (PREMODA) score utilized in this study previously has been outlined in other publications by the Frankfurt Breech at Term Study Group (FRABAT) collective.^5^, ^7^, ^8^, ^9^, ^11^, ^26^ Fetal short‐term morbidity applied if one or more factors of the following occurred: 5 min APGAR <4, fetal birth injury, neurologic deficit, admission to the neonatal intensive care unit for more than 4 days, intubation period >24 h. All cases were investigated in detail. Cases with enhanced fetal morbidity non‐associated with the delivery or delivery mode (e.g., infection, fetal birth defect, hyperbilirubinemia) were excluded (modified PREMODA score, or PREMODA potentially related to delivery mode). Further, manual assistance during vaginal breech birth may become necessary during the delivery of the head or the arms. The applied maneuvers (Louwen maneuver for arm delivery and Frank Nudge for head delivery) used in an upright position were published previously.^5^, ^13^

### 2.4 Statistical analysis

The Kolmogorov–Smirnov test was used to assess whether the variables followed a normal distribution,^27^, ^28^ data not shown. Three types of analysis were utilized: (1) unadjusted bivariate linear regression, (2) unadjusted bivariate nominal‐logistic regression and (3) multivariate adjusted nominal‐logistic regression.

(1) Unadjusted, bivariate linear regression analysis was performed using *t*‐testing. Correlation coefficient, 95% confidence interval and Nagelkerke *r* ^2^ was given and is shown in Table 1.^29^ (2) We carried out an unadjusted bivariate nominal‐logistic regression analysis with the ITD distance as the independent variable. We displayed odds ratio (OR) per unit, 95% confidence interval (CI) and used Wald testing to screen for significant associations in Tables 2 and 3. Primary outcome parameters were cesarean section and fetal short‐term‐morbidity, measured with the modified PREMODA score (for explanation see above). Reverse prediction was performed (see Figure 3). (3) In order to analyze the correlation of the ITD with primary outcome parameters, we performed a multivariate nominal‐logistic regression analysis with adjustment for fetal birth weight, OC and maternal height and displayed OR, 95% CI and *P* value of Wald testing in Table 4. In Table 5, we also performed a multivariate nominal‐logistic regression analysis. Here, we examined a subcohort of vaginal deliveries with the primary outcome parameters fetal short‐term morbidity, assisted arm delivery and assisted head delivery. In this analysis, we adjusted for fetal birth weight, OC and fetal head circumference (see Table 5). Statistical analyses were conducted using JMP software (version 14.0, SAS Institute, Cary, NC, USA), with a *P* value of less than 0.05 deemed statistically significant.

**TABLE 1:** Vaginally intended deliveries (*n* = 876), unadjusted, bivariate linear regression analysis of the ITD in cm.

Variable 2	Correlation (95% CI)	*r* ^2^	*P* value
Maternal age (years)	0.018 (−0.051 to 0.087)	0.000319	0.6111
Maternal height (cm)	0.301 (0.240 to 0.360)	0.090	<0.0001
Maternal weight (kg)	0.191 (0.126 to 0.254)	0.036	<0.0001
BMI	0.068 (0.002 to 0.134)	0.004644	0.0450
OC (cm)	0.167 (0.101 to 0.230)	0.02777	<0.0001

*Note*: BMI, calculated as weight in kilograms divided by the square of height in meters.

Abbreviations: BMI, body mass index; CI, confidence interval; cm, centimeter; ITD, intertuberous distance; OC, obstetrical conjugate.

**TABLE 2:** Outcome variables, unadjusted, bivariate nominal‐logistic regression. Independent variable: ITD (cm).

Dependent variable	Odds ratio per unit (95% CI)	*P* value Wald test
Cesarean section rate	0.73 (0.63–0.84)	<0.001
Epidural analgesia	0.94 (0.81–1.09)	0.400
Postpartum hemorrhage	0.79 (0.50–1.24)	0.313
Transfer to the pediatric ward (*n*, %)	0.96 (0.74–1.24)	0.758
NICU >4 days	0.78 (0.56–1.07)	0.127
Intubation	0.51 (0.23–1.08)	0.088
Fetal birth injury	0.44 (0.16–1.11)	0.093
5 min APGAR <4	0.90 (0.39–1.99)	0.795
Fetal neurologic deficit after discharge	0.35 (0.10–1.09)	0.085
Neonatal infection	1.10 (0.79–1.51)	0.576
PREMODA	0.82 (0.60–1.12)	0.210
Modified PREMODA	0.56 (0.31–0.98)	0.048
Modified PREMODA after exclusion of ITD <11 cm (*n* = 867)	0.62 (0.32–1.12)	0.126

Abbreviations: CI, confidence interval; cm, centimeter; ITD, intertuberous distance; NICU, neonatal intensive care unit.

**TABLE 3:** Subcohort of vaginal deliveries (cesarean sections excluded, *n* = 485), unadjusted, bivariate nominal‐logistic regression, independent variable: ITD (cm).

Variable 2	Odds ratio per unit (95% CI)	*P* value Wald test
Manual assistance	0.77 (0.63–0.94)	0.0126
Help with the delivery of arms	0.94 (0.76–1.17)	0.610
Help with the delivery of the head	0.70 (0.57–0.86)	<0.001
Perineal tear III° + IV°	0.84 (0.46–1.48)	0.549
Transfer to the pediatric ward (*n*, %)	0.93 (0.64–1.34)	0.690
NICU >4 days	0.63 (0.38–1.02)	0.067
Intubation	0.42 (0.18–0.90)	0.032
Fetal birth injury	0.35 (0.13–0.92)	0.040
5 min APGAR <4	0.59 (0.22–1.46)	0.268
Fetal neurologic deficit after discharge	0.14 (0.03–0.57)	0.010
Neonatal infection	1.10 (0.63–1.87)	0.726
PREMODA	0.66 (0.41–1.04)	0.081
Modified PREMODA	0.34 (0.16–0.66)	0.002
Modified PREMODA after exclusion of ITD <11.5 cm	0.43 (0.16–1.00)	0.070

Abbreviations: CI, confidence interval; cm, centimeter; ITD, intertuberous distance; NICU, neonatal intensive care unit.

**TABLE 4:** Adjusted analysis, multivariate nominal‐logistic regression. Independent variable: ITD (cm), adjusted to fetal birth weight, OC and maternal height.

	Odds ratio per unit (95% CI)	*P* value Wald test
Dependent variable: modified PREMODA score		
ITD (cm)	0.67 (0.37–1.19)	0.181
Fetal birth weight (kg)	1.01 (0.25–3.64)	0.991
OC (cm)	0.67 (0.25–1.34)	0.240
Maternal height (cm)	0.94 (0.86–1.03)	0.196
Dependent variable: cesarean section		
ITD (cm)	0.72 (0.62–0.85)	<0.001
Fetal birth weight (kg)	5.14 (3.51–7.64)	<0.001
OC (cm)	0.70 (0.58–0.86)	<0.001
Maternal height (cm)	0.99 (0.97–1.02)	0.740

Abbreviations: CI, confidence interval; cm, centimeter; ITD, intertuberous distance; OC, obstetrical conjugate.

**TABLE 5:** Adjusted analysis of the subcohort of vaginal deliveries (cesarean sections excluded, *n* = 485) multivariate nominal‐logistic regression. Independent variable: ITD (cm), adjusted to fetal birth weight, OC and maternal height. Dependent variable: modified PREMODA score and cesarean section.

	Odds ratio per unit (95% CI)	*P* value Wald test
Dependent variable: modified PREMODA score		
ITD (cm)	0.44 (0.20–0.90)	0.031
Analysis after exclusion of ITD <11.5 cm	0.46 (0.17–1.08)	0.105
Fetal birth weight (kg)	5.6 (0.61–49.8)	0.120
OC (cm)	0.37 (0.12–0.94)	0.053
Fetal head circumference (cm)	1.02 (0.58–1.86)	0.954
Dependent variable: assisted arm delivery		
ITD (cm)	0.90 (0.72–1.13)	0.378
Fetal birth weight (kg)	2.41 (1.16–5.06)	0.018
OC (cm)	0.98 (0.74–1.29)	0.880
Fetal head circumference (cm)	1.05 (0.86–1.27)	0.659
Dependent variable: assisted head delivery		
ITD (cm)	0.67 (0.54–0.83)	<0.001
Analysis after exclusion of ITD <11.5 cm	0.67 (0.52–0.85)	<0.001
Fetal birth weight (g)	1.66 (0.85–3.28)	0.141
OC (cm)	1.18 (0.91–1.51)	0.209
Fetal head circumference (cm)	1.03 (0.96–1.23)	0.761

Abbreviations: CI, confidence interval; cm, centimeter; g, gram; ITD, intertuberous distance; OC, obstetric conjugate.

## 3 RESULTS

**FIGURE 2:**
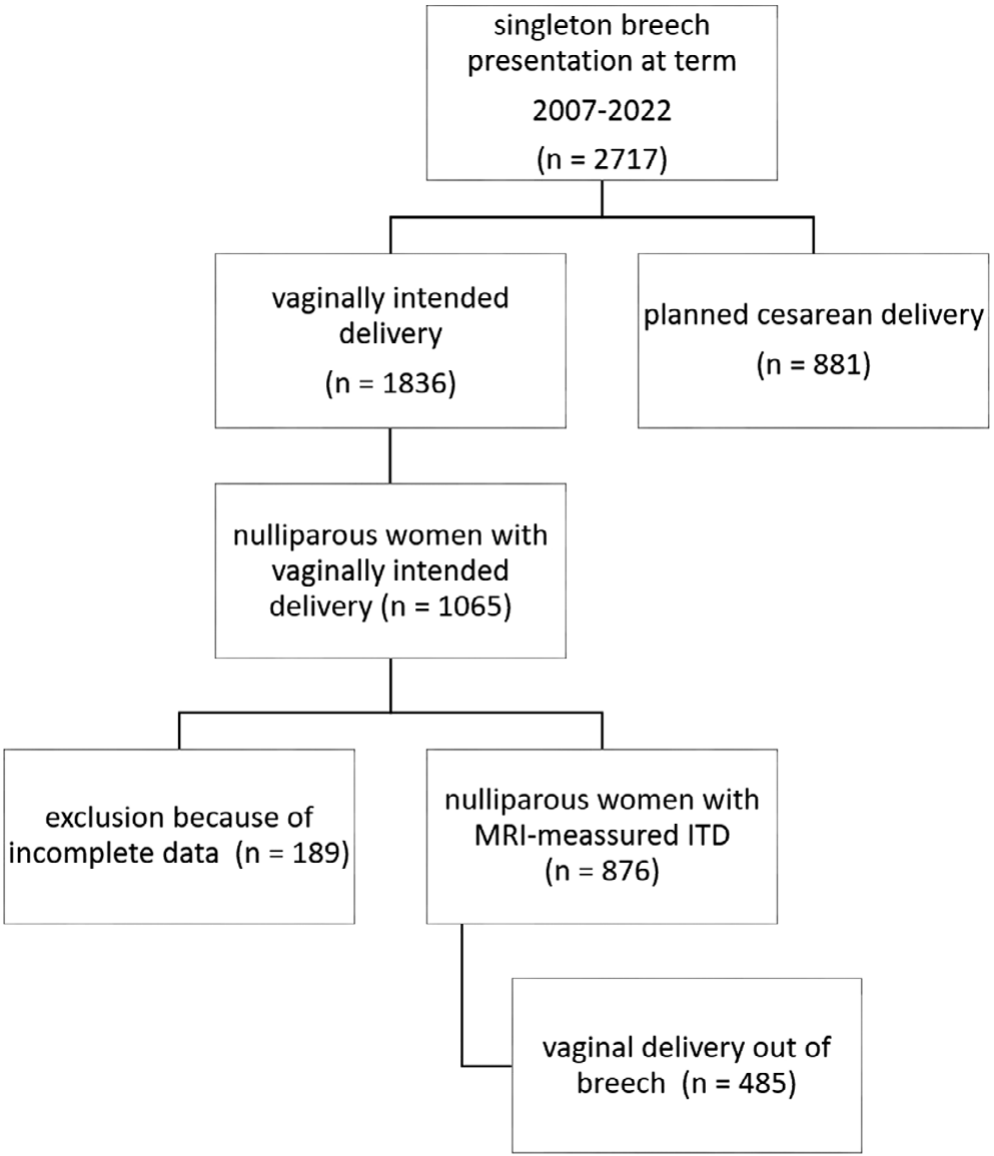
Study cohort flow chart.

A total of 2717 women with a breech presentation at term registered for birth between 2007 and 2022. Of these, 1836 women decided to intend a vaginal delivery. A total of 1065 women were nulliparous and were eligible for our analysis. A total of 189 women had to be excluded because of incomplete data. A total of 876 vaginal intended deliveries were included in our statistical analysis, of which 485 (55.4%) delivered vaginally (Figure 2). Mean age was 31.6 years, the mean body mass index (BMI, calculated as weight in kilograms divided by the square of height in meters) was 22.9, mean duration of pregnancy was 39.5 weeks, mean fetal birth weight was 3.324 kg, mean ITD was 12.8 cm and the total rate of cesarean sections was 44.7%. Table 6 shows further characteristics of the study population.

**TABLE 6:** Characteristics of the study population (*n* = 876).

Characteristics	Values
Maternal age (years, mean ± SD)	31.6 ± 3.8
Maternal height (cm, mean ± SD)	169.2 ± 6.3
Maternal weight (kg, mean ± SD)	65.7 ± 11.5
BMI (mean ± SD)	22.9 ± 3.5
OC (cm, mean ± SD)	12.8 ± 0.8
ITD (cm, mean ± SD)	12.8 ± 0.9
Duration of pregnancy (weeks, mean ± SD)	39.5 ± 1.2
Cesarean section after onset of labor (*n*, %)	392 (44.7%)
Epidural anesthesia (*n*, %)	554 (63.2%)
Manual assistance (*n*, %)	240 (27.4%)
Loss of blood during labor (mL, mean ± SD)	357 ± 206
Hemoglobin on discharge (g/dL, mean ± SD)	10.7 ± 1.4
Fetal birth weight (kg, mean ± SD)	3.324 ± 0.41
Fetal birth length (cm, mean ± SD)	52.3 ± 2.7
Fetal head circumference (cm, mean ± SD)	35.5 ± 1.4

*Note*: BMI, calculated as weight in kilograms divided by the square of height in meters.

Abbreviations: BMI, body mass index; ITD, intertuberous distance; OC, obstetrical conjugate; SD, standard deviation.

The ITD was significantly associated with maternal height (correlation coefficient 0.3, 95% CI: 0.24–0.36, *P* < 0.001), weight (correlation coefficient 0.191, 95% CI: 0.126–0.254, *P* < 0.001) and with the BMI (correlation coefficient 0.068, 95% CI: 0.002–0.134, *P* = 0.045). We found a significant association of the ITD with the OC (correlation coefficient 0.167, 95% CI: 0.101–0.230, *P* < 0.001).

**FIGURE 3:**
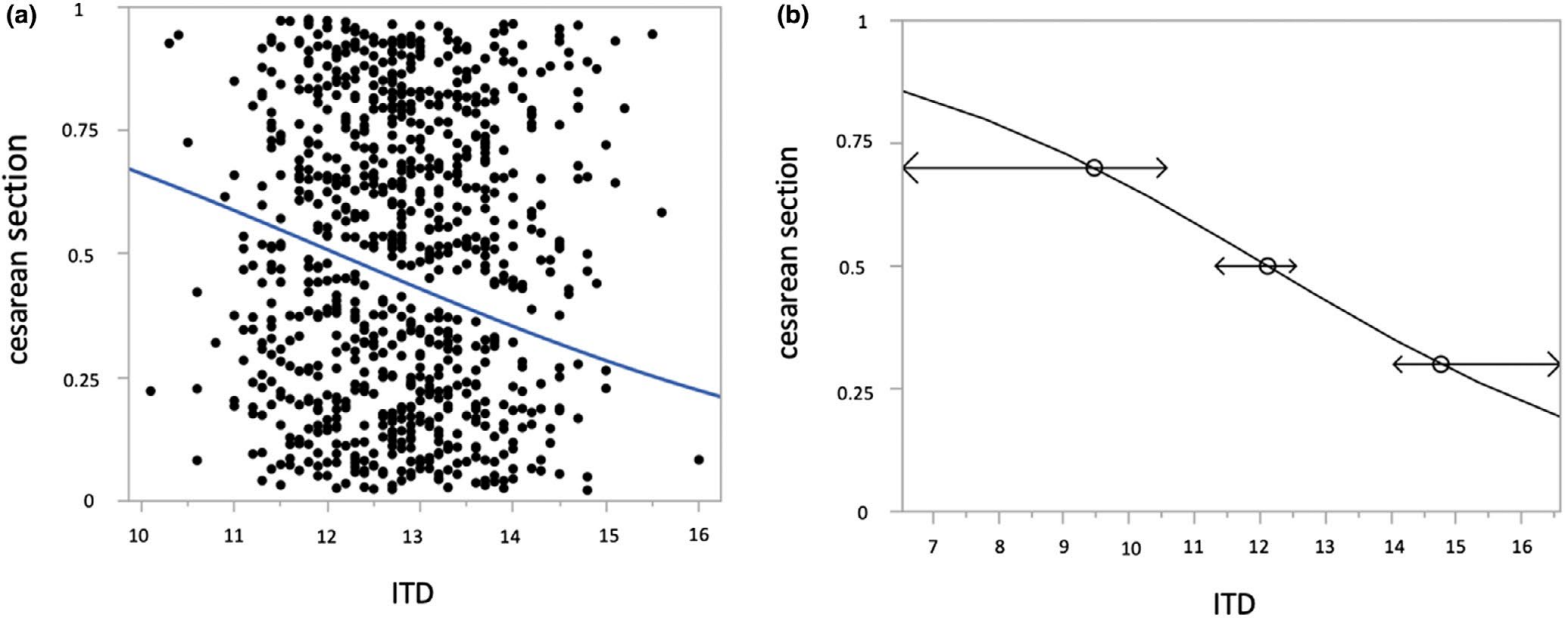
(a) Bivariate, unadjusted nominal‐logistic regression of intertuberous distance (ITD) in centimeters (cm) and cesarean section (CS) rate in vaginally intended breech deliveries (*P* < 0.0001; *r* ^2^ = 0.0148). (b) Inverted prediction of the CS rate depending on the ITD in cm. An ITD of 14.8 cm or less is associated with a 30% likelihood of CS delivery, an ITD decreasing to 12.1 cm or less raises the predicted CS rate to 50%, and an ITD of 9.4 cm or less corresponds to a 70% probability of CS.

Within a bivariate logistic regression analysis of all vaginal intended breech deliveries, a significant negative correlation of the ITD with cesarean section rate (*P* < 0.0001; *r* ^2^ = 0.0148) was found (Figure 3) with an OR of 0.73 per unit (95% CI: 0.63–0.84, Table 2). Inverted prediction analysis disclosed that an ITD of 14.8 cm or below predicts a cesarean section rate of 30%. An ITD below 12.1 cm was associated with a predicted cesarean section rate exceeding 50% (Figure 3). No significant association was found for the ITD and epidural analgesia (OR 0.94, 95% CI: 0.81–1.09, *P* = 0.400) or postpartum hemorrhage (OR 0.79, 95% CI: 0.50–1.24, *P* = 0.313). There was a significant association of the ITD with fetal short‐term morbidity, measured in a combined score, the modified PREMODA score (OR 0.56, 95% CI: 0.31–0.98, *P* = 0.048). However, when all cases with an ITD <11 cm (*n* = 9) were excluded, the association between the ITD and the modified PREMODA score was no longer significant (OR 0.62, 95% CI: 0.32–1.12, *P* = 0.126). Table 2 shows further characteristics of the logistic regression analysis of all vaginally intended breech deliveries and all parameters leading to the mod. PREMODA score (4‐min APGAR <4, intubation, stay on the NICU >4 days, neurologic deficit at discharge).

Because maternal height was highly significantly associated with the ITD and the OC as well as fetal birth weight are known and important confounders for delivery outcome, we performed multivariate analyses (Table 4). The modified PREMODA score showed no association with the ITD (OR per unit 0.67, 95% CI: 0.37–1.19, *P* = 0.181) when adjusted to fetal birth weight, OC and maternal height (Table 4). Cesarean section was significantly associated with the ITD (OR per unit 0.72, 95% CI: 0.62–0.85, *P* < 0.001) after adjustment (Table 4).

In order to examine the ITD and its impact on vaginally ended deliveries and manual assistance, we performed a subcohort analyses on all vaginal deliveries (*n* = 485). Unadjusted nominal regression analysis showed a significant association of the ITD with manual assistance (OR per unit 0.77, 95% CI: 0.63–0.94, *P* = 0.0126), assisted head delivery (OR per unit 0.70, 95% CI: 0.57–0.86, *P* < 0.001). There was no significant association of the ITD and high grade perineal tear (OR per unit 0.84, 95% CI: 0.46–1.34, *P* = 0.690). Fetal short‐term morbidity was significantly associated with the ITD (OR per unit 0.34, 95% CI: 0.16–0.66, *P* = 0.002). When excluding deliveries with an ITD of below 11.5 cm, significance of this correlation vanished (OR per unit 0.43, 95% CI: 0.16–1.00, *P* = 0.070) (see Table 3).

For the subcohort of vaginal deliveries, the following confounding variables were found: fetal birth weight, OC and fetal head circumference.

A multivariate regression analysis, adjusted to fetal birth weight, OC and the fetal head circumference showed a significant association of the ITD with fetal short‐term morbidity (OR per unit 0.44, 95% CI: 0.20–0.90, *P* = 0.031) which significance vanished after excluding deliveries with an ITD of below 11.5 cm (*n* = 63) (OR per unit 0.46, 95% CI: 0.17–10.8, *P* = 0.105) (see Table 5).

Assisted arm delivery was not associated with the ITD in multivariate adjusted analysis (OR per unit 0.9, 95% CI: 0.72–1.13, *P* = 0.378). The analysis revealed a significant association of fetal birth weight and assisted arm delivery (OR per unit 2.41, 95% CI: 1.16–5.06, *P* = 0.018). Assisted head delivery and the ITD correlated significantly with adjusted multivariate analysis (OR 0.67, 95% CI: 0.54–0.83, *P* < 0.001). This effect was still significantly shown after exclusion of deliveries with an ITD of below 11.5 cm (OR per unit 0.67, 95% CI: 0.52–0.85, *P* < 0.001) (see Table 5).

## 4 DISCUSSION

To date, guidelines on vaginal breech delivery have several restrictions concerning estimated fetal birth weight, maternal parity, and other feto‐maternal parameters but disregard the influence of prenatal pelvic measurements despite contradicting evidence. Data from several studies evince an influence of pelvic measurements on birth outcome parameters.^12^, ^14^, ^20^, ^22^, ^23^, ^26^, ^30^, ^31^, ^32^, ^33^, ^34^ There is a high demand for additional evidence on parameters that predict delivery outcomes to enhance patient counseling quality regarding the safest delivery mode out of the breech presentation as well as success rates in terms of a vaginal birth. As far as we are aware, this is the largest study to analyze the impact of the ITD measured by MRI on birth outcome parameters in nulliparous women with breech presentation at term. The ITD is the shortest osseous diameter in the pelvic outlet, where during head delivery, the biparietal diameter of the fetal head has to pass. Notably, pelvic measurements correlate with each other, such as the pubic arch angle or the interspinous distance.^12^, ^31^ Results on analyses of the pubic arch angle did resemble the results presented in this study but did not reach statistical significance (data not shown).

Given the relevance of pelvic outlet dimensions to delivery mechanics, the delivery technique itself also plays a crucial role. At our study center, deliveries are performed in an upright maternal position. This facilitates a hands off delivery, whereas in a supine position (mother on her back), always at least one maneuver (e.g., Bracht) has to be performed to deliver the baby. When there is a complication, like a delayed development of the fetal head or shoulders/arms, the previously described maneuvers in upright maternal position are performed: The Louwen maneuver to support arm and shoulder delivery and the Frank Nudge to aid head development.^5^, ^13^ This monocentric cohort study examined the influence of the ITD on perinatal outcome parameters in vaginal breech deliveries. For this purpose, we investigated fetal short‐term outcome parameters as well as the requirement of manual assistance during head or arm delivery in vaginally intended breech deliveries at term in nulliparous women. The vaginal delivery rate was 485 out of 876 (55.4%), with manual assistance required in 240 out of 485 successful vaginal deliveries (49.5%). We found a significant negative correlation between the ITD and cesarean section rate (Figure 3). Inverted prediction analysis disclosed a probability of 50% for cesarean section with an ITD of 12.1 cm or below. These findings all together suggest a clear influence of the ITD on birth mechanics as the narrowest osseous “bottleneck” of the pelvic outlet. A short ITD was significantly associated with fetal morbidity, but after excluding patients with an ITD of less than 11 cm, the significant association disappeared. The effect was not consistent since in a multivariate analysis with adjustment to fetal birth weight, the OC and maternal height no significant association of the ITD and fetal short term morbidity was detected. Within our center, deliveries with an ITD of below 11 cm were excluded after Klemt et al. data were published to avoid complicated head deliveries.^12^ Here, we publish data on deliveries before and after this exclusion criterion was found and implemented into patient's counseling. The fact that the association of fetal short‐term morbidity and the ITD is dependent on the in‐ or exclusion of pregnant women with an ITD below 11 cm underlines the adjusted recommendation. In vaginal deliveries and the respective subcohort analysis, multivariate adjusted regression showed an independent association of the ITD and fetal short‐term morbidity as well as assisted head delivery. When excluding deliveries with an ITD of below 11.5 cm, significance level was not reached (Table 5). This clearly demonstrates that the ITD, especially when it is narrow, often is not an obstacle for the fetal body but could be for the after coming head. The likelihood of birth injury, neurologic deficiency, and the need for intubation of the newborn were not associated with the ITD. Pregnant women should be told about the probability for manual assistance during breech delivery, also in relation to their individual pelvimetric measurements in order to be able to make an informed decision on their preferred delivery mode.

Our results clearly show the value of prenatal pelvimetry and appropriate patient selection. There is evidence that peripartum fetal morbidity might be reduced by excluding women with an ITD of below 11 cm from vaginal birth attempts, as it is done in the study center. Since the sample size of deliveries with an ITD of below 11 cm is very low in our cohort, more data on deliveries with a low ITD are needed in order to generate sufficient evidence for a general recommendation. It should be noted that the used morbidity score is adapted from the study by Goffinet et al. and measures short‐term morbidity.^26^ Long‐term follow up data on cases with a positive PREMODA score show, that the respective newborns predominantly do not suffer from long‐term consequences such as an impaired neurologic development.^15^

Lia et al. found an association between the ITD and the duration of the second stage of labor.^23^ We came to the result that the ITD impacts the need for obstetric maneuvers, emphasizing the shown effect on the course of labor and delivery. In light of our here published data and the available data from the literature, it seems evident that a narrow pelvic outlet can lead to an arrest in or delayed fetal head delivery, subsequently leading to enhanced short‐term fetal morbidity. As a result, patients have to receive an appropriate examination prior to labor and counseling addressing pelvic measurements when a breech presentation is present at term.

Given the limited feasibility of MRI in certain clinical settings, sonographic methods provide a practical and accessible alternative for intrapartum pelvic assessment. Recent studies have highlighted the potential of these methods in predicting delivery outcomes in cases of vaginal head deliveries. Liang and Gao investigated the pubic arch angle (PAA), which, similar to the ITD, reflects the pelvic outlet and was found to be a significant positive predictor of both vaginal delivery and the duration of the second stage of labor.^31^ Supporting this, computed tomography pelvimetry studies have demonstrated that the PAA correlates significantly with both the ISD and the OC, reinforcing its value as a reliable proxy for pelvic dimensions.^34^ Complementing these findings, MRI pelvimetry has further highlighted the predictive value of pelvic measurements in vaginal head deliveries. Li et al. measured the ITD by MRI and found that the cephalopelvic index of diameter—a composite of ITD, interspinous diameter, and OC—was the main predictor of successful vaginal head delivery.^32^ Similarly, Gowri et al. reported that ITD contributed to the outlet index, which was larger in women who delivered vaginally, emphasizing its role in predicting birth outcomes.^33^

Nonetheless, a key limitation of our study was the potential for selection bias, since all data were sourced from a single center. Women who opted for the vaginal birth approach were strongly preselected by our obstetricians during counseling and were, therefore, highly motivated. Another potential bias of our study was the medical system in a high‐resource country and clinic. An MRI before labor does not seem feasible, especially for a setting in developing countries, although the ITD can also be measured with a clinical examination. In order to validate and compare MRI‐derived measurements, comparative studies are needed to implement routine pelvic examination prior to breech delivery without the necessity of an MRI imaging. The present study does not implement other fetal body measurements and does not include sonographic estimation, which would generate valuable input on the counseling process in breech presentation.

## 5 CONCLUSION

In this study, we were able to demonstrate that while overall morbidity is low, a narrow ITD is associated with fetal short‐term morbidity, which could be avoidable when pregnant women with an ITD of below 11 cm receive a recommendation for a cesarean section. Additionally, we were able to show the influence of the ITD on cesarean section rate and the need for obstetric maneuvers during vaginal breech delivery, especially during the development of the head. This evidently shows the benefit of prenatal pelvimetry in breech presentation at term.

## AUTHOR CONTRIBUTIONS

**Julia Schmidt:** Conceptualization, data curation, investigation, methodology, writing – original draft, visualization, project administration. **Lukas Jennewein:** Conceptualization, methodology, supervision, writing—original draft, writing—review and editing, resources. **Frank Louwen:** Supervision, writing—review and editing. **Anna Elisabeth Hentrich, Anne Kristina Kämpf, Eileen Deuster, Samira Catharina Hoock:** Data curation, writing—review and editing. All authors have reviewed and approved the final version of the manuscript.

## FUNDING INFORMATION

No funding was acquired.

## CONFLICT OF INTEREST STATEMENT

The authors have no conflicts of interest.

## TRIAL REGISTRATION

DRKS00025030, Deutsches Register klinischer Studien, https://drks.de/search/de/trial/DRKS00025030/details.

## Data Availability

Research data are not shared.
